# Evaluation of a direct method for detecting extended-spectrum β-lactamase using the Cica-beta test on positive blood culture bottles

**DOI:** 10.1128/spectrum.01112-25

**Published:** 2025-10-08

**Authors:** Tomohide Okinaka, Yuji Teshima, Yoshimi Furuno, Miho Takeishi, Ayami Tashiro, Yoko Matsuda, Ayumi Irie, Tomomi Marutani, Hidenobu Koga, Takashi Matono

**Affiliations:** 1 Department of Infectious Diseases, Aso Iizuka Hospital 13750 , Fukuoka, Japan; 2 Department of Microbiology Laboratory, Aso Iizuka Hospital 13750 , Fukuoka, Japan; 3 Infection Control Center, Aso Iizuka Hospital 13750 , Fukuoka, Japan; 4 Department of Clinical Research Support Office, Aso Iizuka Hospital 13750 , Fukuoka, Japan; 5 Division of Infectious Disease and Hospital Epidemiology, Saga University Hospital 721831, Saga, Japan; University of Maryland School of Medicine, Baltimore, Maryland, USA

**Keywords:** bloodstream infection, Cica-beta test, rapid diagnosis, extended-spectrum β-lactamase

## Abstract

The global prevalence of extended-spectrum β-lactamase (ESBL)-producing Enterobacterales is a growing concern, underscoring the need for rapid detection to enable timely, targeted therapy. Conventional methods for ESBL detection, typically involving subculture followed by phenotypic screening and confirmatory tests based on the Clinical and Laboratory Standards Institute guidelines, require extended turnaround times. While rapid molecular diagnostic tests exist, their availability can be limited in some settings due to cost or infrastructure requirements, whereas matrix-assisted laser desorption/ionization time-of-flight mass spectrometry for bacterial identification is more widely adopted. The chromogenic cephalosporin HMRZ-86-based Cica-beta test is a simpler alternative but traditionally requires colonies from subcultures. This study evaluated a direct Cica-beta test using positive blood cultures for rapid extended-spectrum β-lactamase detection. Using bacterial pellets prepared from 109 positive blood cultures of *Escherichia coli*, *Klebsiella pneumoniae*, *Klebsiella oxytoca*, or *Proteus mirabilis*, the direct Cica-beta test demonstrated high sensitivity (96.3%, 95% confidence interval [CI]: 81.0–99.9) and specificity (100%, 95% CI: 93.5–100), with a positive predictive value of 100% (95% CI: 81.0–100) and a negative predictive value of 98.8% (95% CI: 93.5–100), when compared to conventional methods. This direct method provides a faster alternative to traditional subculture-based testing, enabling prompt antibiotic adjustments. Although challenges remain, such as the potential misclassification of certain β-lactamases, such as AmpC, this approach offers a promising, cost-effective strategy for rapid diagnosis. It has the potential to improve patient outcomes by facilitating timely and appropriate antimicrobial therapy. Further multicenter studies are necessary to validate these findings.

**IMPORTANCE:**

Extended-spectrum β-lactamase-producing Enterobacterales pose significant challenges in clinical practice due to their resistance to β-lactam antibiotics and association with increased mortality in bloodstream infections. Rapid and accurate identification of these organisms remains crucial for guiding initial antimicrobial therapy and improving patient outcomes. Conventional testing methods are time-consuming, and advanced molecular techniques, such as FilmArray, are costly and not universally available. This study evaluated a novel application of the chromogenic cephalosporin HMRZ-86-based Cica-beta test, a direct method using bacterial pellet from positive blood cultures for extended-spectrum β-lactamase detection. It demonstrated high diagnostic accuracy, with sensitivity and specificity comparable to conventional and FilmArray methods, while significantly reducing result turnaround time. This cost-effective, rapid diagnostic tool offers practical advantages in diverse clinical settings and resource-limited environments. Its implementation has the potential to enhance antimicrobial stewardship programs, support timely clinical decision-making, and aid efforts to combat antibiotic resistance.

## INTRODUCTION

Extended-spectrum β-lactamase (ESBL)-producing bacteria can hydrolyze a wide range of β-lactam antibiotics, including penicillins, cephalosporins, and monobactams, which complicates the treatment of infections caused by these organisms. These bacteria primarily belong to the order *Enterobacterales*, and the Clinical and Laboratory Standards Institute (CLSI) guidelines recommend testing for ESBL production in four species: *Escherichia coli*, *Klebsiella pneumoniae*, *Klebsiella oxytoca*, and *Proteus mirabilis* (1). Recently, the global prevalence of ESBL-producing bacteria has increased (2, 3), presenting a significant challenge in clinical settings. In Japan, CTX-M-type β-lactamases are the most common ESBLs (4). The presence of ESBL-producing bacteria in blood cultures can limit treatment options, often complicating therapy. Furthermore, bloodstream infections caused by ESBL-producing bacteria are associated with higher mortality rates compared to those caused by non-ESBL-producing organisms (5, 6). Rapid and appropriate antibiotic therapy is crucial for improving patient outcomes in such cases (7–10).

Although carbapenem-resistant *Enterobacterales* and AmpC-producing bacteria can cause refractory bloodstream infections, ESBL-producing bacteria are more commonly encountered in outpatient or emergency room settings. Early identification of ESBL-producing bacteria can help prevent the unnecessary use of carbapenems, thereby reducing the risk of promoting the proliferation of carbapenemase-producing antibiotic-resistant strains. Carbapenem use has been implicated in the emergence of carbapenemase producers and the development of other resistance mechanisms, including reduced outer membrane porin expression, genetic mutations, overproduction of multidrug efflux pumps, and alterations in penicillin-binding proteins (11). The introduction of rapid diagnostic technologies is essential to improving patient outcomes, preventing the development of resistance, and offering considerable public health benefits.

The primary limitation of existing diagnostics is the turnaround time. Conventional phenotypic methods recommended by the CLSI, such as screening and confirmatory disk tests, are reliable but require subculture and typically take 48–72 hours to yield a final result (1). While advanced technologies have been developed to shorten this timeframe, they each have practical limitations. Matrix-assisted laser desorption/ionization time-of-flight mass spectrometry (MALDI-TOF MS) has revolutionized the speed of bacterial species identification, but it cannot directly detect resistance mechanisms like ESBL production (12). Concurrently, rapid molecular panels can detect common resistance genes (e.g., *bla*CTX-M) within an hour of a positive culture, but their high cost has limited their universal adoption.

This diagnostic gap highlights the need for a rapid, accurate, and cost-effective method for ESBL detection. The chromogenic cephalosporin-based Cica-beta test is a simple strip-based assay, but it traditionally requires colonies from a subculture (13–16). Although recent CLSI guidelines have de-emphasized routine ESBL confirmation for susceptibility reporting, early identification remains clinically valuable for guiding initial therapy, especially in critical infections like bacteremia (1). Therefore, this study aimed to evaluate the diagnostic accuracy of a direct Cica-beta test method using bacterial pellets prepared directly from positive blood culture bottles.

## MATERIALS AND METHODS

### Blood culture sample collection and bacterial identification

This prospective cohort study was conducted at Iizuka Hospital in Iizuka City, Fukuoka Prefecture, Japan. The hospital is a tertiary care institution with 1,048 beds, collecting 15,000–18,000 blood culture sets annually (typically one set consisting of one aerobic and one anaerobic bottle), of which 1,800–2,000 sets test positive. This prospective cohort study included positive blood culture bottles collected between April and August 2024 at Iizuka Hospital. Inclusion criteria were (i) detection of gram-negative bacilli by Gram staining performed on the positive blood culture fluid, and (ii) direct identification of one or more of the four target bacterial species (*E. coli*, *K. pneumoniae*, *K. oxytoca*, or *P. mirabilis*, for which the CLSI defines ESBL screening criteria [1]) using the MALDI Biotyper mass spectrometry system (Bruker Daltonics Inc., Billerica, MA, USA) directly from the positive blood culture broth. Exclusion criteria were (i) identification of bacterial species other than the four target species by the MALDI Biotyper, or (ii) Gram stain findings suggesting polymicrobial infection involving non-target species (although cases with mixtures only consisting of the four target species were included, as described later), or (iii) duplicate samples from the same patient within a defined timeframe. The blood culture bottles used in this study were primarily BD BACTEC Plus Aerobic medium in plastic culture vials, BD BACTEC Lytic Anaerobic medium in plastic culture vials, and BD BACTEC Peds Plus/F culture vials (Becton, Dickinson and Company, Franklin Lakes, NJ, USA).

### Preparation of bacterial pellets for the Cica-beta test

A bacterial pellet was prepared from positive blood culture fluid and used as the sample for the Cica-beta test using the MALDI Sepsityper kit (Bruker Daltonics Inc.), a blood culture pretreatment reagent kit (Bruker Daltonics Inc.). One milliliter of positive blood culture fluid was transferred to a reaction tube, and 200 µL of lysis buffer was added. The mixture was vortexed for 10 seconds. The resulting sample was then centrifuged using a Centrifuge 5425 (Eppendorf SE, Hamburg, Germany) with a rotor at 21,300 × *g* for 2 minutes at room temperature (approximately 25°C), and the upper layer of the mixture was discarded. Next, 1 mL of washing buffer was added to the pellet, which was resuspended by pipetting. A second centrifugation was performed at 21,300 × *g* for 1 minute at room temperature. Subsequently, the supernatant was discarded, and the resulting bacterial pellet was used for testing. If blood components were visually confirmed in the bacterial pellet, the steps involving the addition of washing buffer and centrifugation were repeated to remove the contaminants.

### Cica-beta direct test and colony test

**Fig 1:**
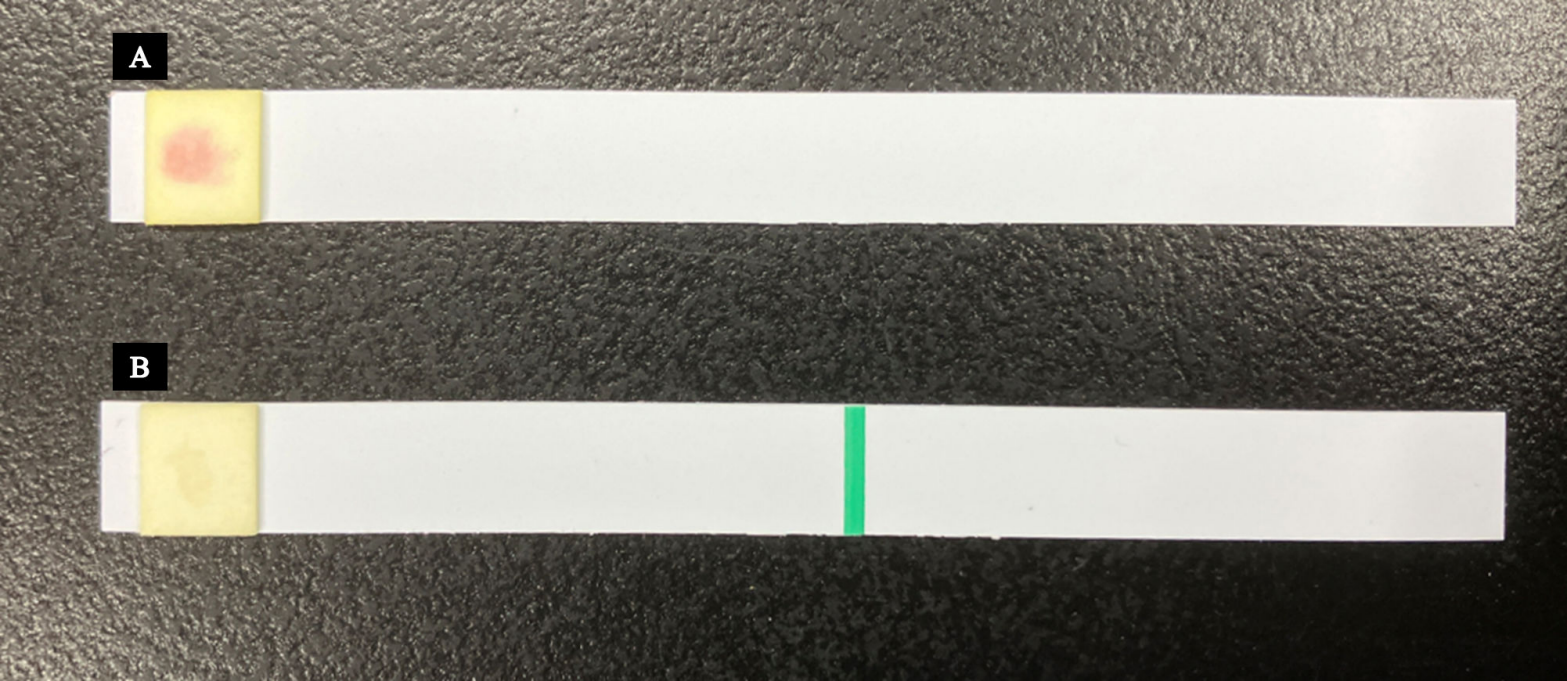
Detection of ESBL-producing bacteria using Cica-beta test strips. Two types of Cica-beta test strips (Kanto Chemical Co., Inc., Tokyo, Japan) were used to detect ESBL activity: Cica-beta test I (**A**; without an inhibitor) and Cica-beta test CVA (**B**; containing CVA). A chromogenic substrate (HMRZ-86) was applied to the filter paper on each strip, which underwent a color change to red when hydrolyzed by ESBL. Bacterial samples were applied to the test strips using a toothpick, followed by incubation at room temperature (approximately 25°C) for 15 min. A red color change in the Cica-beta test I (**A**), but not in the Cica-beta test CVA (**B**), confirmed ESBL production (CVA inhibited ESBL activity); no color change on either strip indicated that the strain was non-ESBL producing.

Two types of Cica-beta test strips (Kanto Chemical Co., Inc., Tokyo, Japan) were used in this study: (i) Cica-beta test I, which did not contain an inhibitor, and (II) Cica-beta test containing clavulanic acid (CVA). A single drop of chromogenic substrate solution containing HMRZ-86 was applied to the filter paper section of each test strip, which turned red when specifically degraded by ESBL. A small amount of the bacterial pellet, sufficient to be visible on the tip, was collected using a toothpick. This sample was then evenly applied to the filter paper section of the test strip by firmly rubbing the toothpick tip onto the surface. The strips were left to react at approximately 25°C for 15 minutes, after which the results were assessed based on color changes. If the substrate solution on the Cica-beta test I strip was degraded, resulting in a red color change, and no color change was observed on the Cica-beta test CVA strip, it was determined that ESBL activity was inhibited by CVA, and the strain was identified as ESBL producing (Fig. 1). The absence of a red color change on both test strips indicated that the strain was not ESBL producing. Colonies obtained through subculture were tested for ESBL production using both types of Cica-beta test strips, following the same procedure as the Cica-beta Direct test.

### Fully automated multi-parameter genetic testing FilmArray blood culture panel for bacterial species identification and detection of resistance genes

A 0.2 mL sample was extracted from positive blood culture bottles and tested using the BioFire Blood Culture Identification Panel 2 (BCID2; bioMérieux SA, Marcy-l'Étoile, France) in accordance with the manufacturer’s instructions. The criteria for interpretation relevant to this study were the detection of *E. coli*, *K. pneumoniae*, *K. oxytoca*, or *P. mirabilis* along with the *CTX-M* gene group (the primary ESBL gene target of this panel), indicating likely ESBL production. The absence of *bla*CTX-M in the presence of these species suggested a non-ESBL producer according to this specific target. The panel also detects other resistance genes (e.g., carbapenemase genes), the results of which were recorded.

### Confirmatory testing

The performance of the direct Cica-beta test was evaluated against the conventional CLSI-recommended workflow, which serves as the reference standard in this study. This entire conventional workflow, from a positive blood culture signal to a final confirmed result, typically requires 48–72 hours and involves the following steps. First, colonies obtained from subcultures of the positive blood culture fluid were subjected to ESBL screening using antimicrobial susceptibility testing (broth microdilution method). This was performed with the MicroScan WalkAway 96 plus microbial identification and susceptibility analysis system (Beckman Coulter, Inc., Brea, CA, USA) and MicroScan Neg EN MIC 2 J or 3 J panels (Beckman Coulter, Inc.). The CLSI-based screening criteria were as follows: for *E. coli*, *K. pneumoniae*, and *K. oxytoca*, an MIC of ≥8 µg/mL for cefpodoxime (CPDX), ≥2 µg/mL for ceftazidime (CAZ), ≥2 µg/mL for aztreonam, ≥2 µg/mL for cefotaxime (CTX), or ≥2 µg/mL for ceftriaxone; and for *P. mirabilis*, a MIC of ≥2 µg/mL for CPDX, ≥2 µg/mL for CAZ, and ≥2 µg/mL for CTX. Any strain meeting these criteria was classified as screening positive. Second, to confirm these screening-positive strains, testing was conducted using the disc diffusion method with ESBL confirmation disks containing CTX (30 µg) or CAZ (30 µg) combined with CVA. The specific disks used were “ESBLs-CTX/CVA ‘Eiken’” and “ESBLs-CAZ/CVA ‘Eiken’,” both manufactured by Eiken Chemical Co., Ltd. (Tokyo, Japan).

### Data analysis

The diagnostic performance of the direct Cica-beta test (sensitivity, specificity, predictive values, and likelihood ratios) was evaluated against the reference standard. The reference standard for ESBL determination was defined as the CLSI-recommended phenotypic confirmatory test using disk diffusion, as described above. The results of the direct Cica-beta test were also compared with those obtained from the FilmArray Blood Culture Panel (BioFire) and the Cica-beta test performed on colonies subcultured from the positive blood culture fluid (colony Cica-beta test) to assess concordance. A 2 × 2 contingency table was used to calculate sensitivity, specificity, positive predictive value (PPV), negative predictive value (NPV), positive likelihood ratio (PLR), negative likelihood ratio (NLR), and 95% confidence interval (CI) were calculated using a 2 × 2 contingency table. Fisher’s exact test was used for categorical variable comparisons, and Cohen’s kappa coefficient was calculated. Statistical analyses were performed using R version 3.4.1 (R Foundation for Statistical Computing, Vienna, Austria).

### Patient information

The following variables were extracted from the medical records: age, sex, whether the patient was receiving antimicrobial agents at the time of blood culture collection, details of such treatments, whether the patient was receiving immunosuppressants, whether the infection was community acquired or hospital acquired, whether the patient was in the intensive care unit, time from infection onset to blood culture collection, the quick Sequential Organ Failure Assessment (qSOFA) score, Pitt bacteremia score, and Charlson Comorbidity Index.

## RESULTS

### Patient background

The baseline characteristics of the patients are summarized in Table S1. The median age of the patients was 81 years, and 55 patients were male. At the time of blood culture collection, 18 patients were receiving antimicrobial therapy, with 10 receiving beta-lactam antibiotics and six receiving sulfamethoxazole-trimethoprim or atovaquone for the prevention of *Pneumocystis* pneumonia. Seventeen patients were administered immunosuppressive drugs. Twenty-four patients had nosocomial infections, and five patients were admitted to the intensive care unit. Blood cultures were obtained from 92 patients within 24 hours of symptom onset. The median qSOFA score was 1, the median Pitt bacteremia score was 1.5, and the median Charlson Comorbidity Index was 3.

### Diagnostic accuracy of the direct Cica-beta test

**Fig 2:**
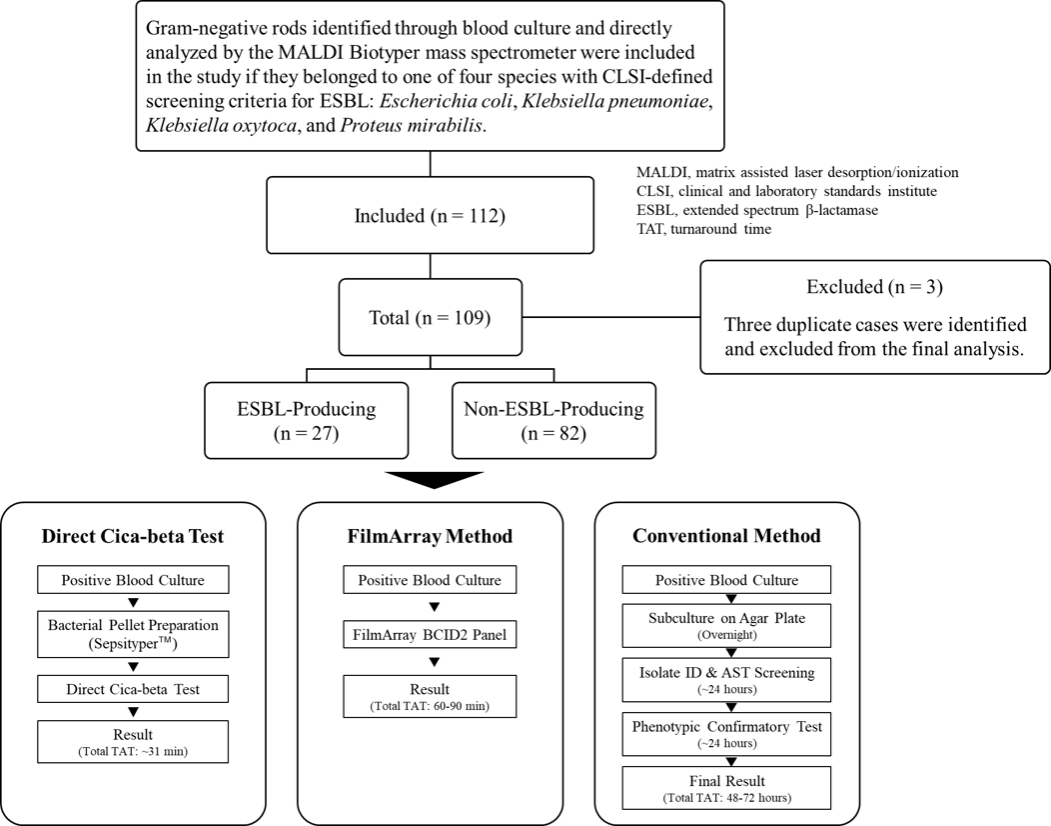
Study flowchart illustrating patient enrollment and parallel diagnostic workflows. The flowchart details the screening and inclusion process, starting from all positive blood cultures identified during the study period, leading to the final cohort of 109 cases. The final cohort is broken down by the reference standard result (ESBL producing or non-ESBL producing). The three parallel diagnostic pathways evaluated for the final cohort are shown at the bottom, comparing the key steps and total turnaround time (TAT) for the direct Cica-beta method, the molecular method (FilmArray BCID2), and the conventional reference method.

A total of 112 patients were included in the study. Three duplicate cases were excluded, resulting in a final study population of 109 cases (Fig. 2). Due to supply adjustments of BD BACTEC Blood Culture Bottles during the study, 14 of 109 cases had only one set of blood cultures collected. The bacterial species isolated were *E. coli* in 84 cases (25 of which were ESBL producing), *K. pneumoniae* in 16 cases (one was ESBL producing), *K. oxytoca* in two cases, *P. mirabilis* in five cases, *E. coli* (non-ESBL) + *K. oxytoca* (ESBL-producing) in one case, and *K. pneumoniae + K. oxytoca* in one case (Table 1).

**TABLE 1:** Distribution of isolated bacterial species

Bacterial species	Total cases (*n*)	ESBL-producing cases^*a*^ (*n*)
*E. coli*	84	25
*K. pneumoniae*	16	1
*K. oxytoca*	2	0
*P. mirabilis*	5	0
*E. coli* (non-ESBL) *+ K. oxytoca* (ESBL)	1	1
*K. pneumoniae + K. oxytoca*	1	0
Total	109	27

^*a*^ ESBL-producing status was determined by the CLSI phenotypic confirmatory test (disk diffusion).

Among the 27 isolates confirmed as ESBL-producers, all 27 (100%) were resistant to ceftriaxone, and 48.1% (13/27) were resistant to ceftazidime. In contrast, among the 82 non-ESBL-producing isolates, none were resistant to either ceftriaxone or ceftazidime. This non-ESBL group did, however, include one isolate (1.2%, 1/82) identified as an AmpC β-lactamase hyperproducer. Importantly, no carbapenemase-producing isolates, including metallo-β-lactamase producers, were detected phenotypically in our cohort. This finding was consistent with the molecular results, as the FilmArray BCID2 panel did not detect any of its targeted carbapenemase genes (*bla*KPC, *bla*NDM, *bla*VIM, *bla*OXA-48-like, or *bla*IMP) in the tested samples. An analysis of the antimicrobial treatment regimens for the 27 patients with ESBL-producing bacteremia revealed that 40.7% (11/27) had received an initial empiric antibiotic that was not active against their isolate, necessitating a subsequent change to an effective therapy.

The detection of ESBL-producing bacteria by the direct Cica-beta test was consistent with the FilmArray results for all 109 cases (100%). The results of the direct Cica-beta test were also consistent with those of the colony and conventional confirmatory testing methods in 108 of 109 cases (99.1%). The sensitivity and specificity of the direct Cica-beta test method were 96.3% (95% CI: 81.0–99.9) and 100% (95% CI: 93.5–100), respectively (Table 2). The PPV was 100% (95% CI: 81.0–100), and the NPV was 98.8% (95% CI: 93.5–100). The PLR was infinite, NLR was 0.037 (95% CI: 0.005–0.253), and Cohen’s kappa coefficient was 0.975 (95% CI: 0.926–1.024). The only case of disagreement involved a mixed culture identified as *E. coli* (non-ESBL) + *K. oxytoca* (ESBL-producing by confirmatory testing) + *K. oxytoca* (ESBL-producing by confirmatory testing). This ESBL production was not detected by the direct Cica-beta test, the colony Cica-beta test, or the FilmArray panel (which did not detect *bla*CTX-M). Further analysis of the isolated *K. oxytoca* (ESBL-producing) strain was performed using the Cica Genius ESBL Genotype Detection Kit 2 (Kanto Chemical Co., Inc.), which targets major ESBL genotypes including CTX-M-1 group, CTX-M-2 group, CTX-M-9 group, and certain TEM-type and SHV-type ESBLs. However, none of these targeted genotypes were detected.

**TABLE 2:** Result of ESBL evaluation employing both the Cica-beta direct test and confirmatory testing method

		Confirmatory testing method^*a*^	
		ESBL	Non-ESBL
Cica-beta direct method	ESBL	26	0
	Non-ESBL	1	82

^*a*^ Confirmatory testing was conducted using disk diffusion to determine the presence or absence of ESBL.

## DISCUSSION

In this study, we evaluated the diagnostic accuracy of the direct Cica-beta test, which uses a bacterial pellet prepared from positive blood cultures. When compared with the conventional double-disk method as the gold standard, this test demonstrated high sensitivity (96.3%) and specificity (100%) for detecting ESBL-producing bacteria. These results are encouraging, especially when considering that previous studies evaluating the HMRZ-86-based Cica-beta test on colonies reported variable and sometimes lower sensitivities (e.g., ranging from 76.5% to 100%) (13–15). Several factors may have contributed to the high sensitivity observed in our direct application. Potential reasons could include enhanced enzyme activity detection from the concentrated bacterial pellet prepared directly from broth culture, as opposed to colonies from agar plates. Additionally, our protocol’s effective removal of blood components likely improved the clarity of the colorimetric reaction. This is a critical point, as Jain et al. previously reported that direct testing on centrifuged supernatants could be affected by the presence of blood components, which can influence colorimetric interpretation (17). The additional washing and hemolysis steps performed in our study when blood components were visible were likely key to overcoming this challenge and achieving reliable results.

Several rapid methods for ESBL detection directly from positive blood cultures have been developed to expedite diagnosis. For example, the ESBL Nordmann/Dortet/Poirel test (INSERM, Paris, France), a biochemical assay, reported a sensitivity and specificity of 100% each when applied directly to positive blood cultures, with results within 30 minutes (18). Boattini et al. evaluated several methods directly from blood cultures (19): the Rapid ESBL NP test (Liofilchem, Roseto degli Abruzzi, Italy) showed a sensitivity of 87.5% and specificity of 97.8%, while the NG-Test CTX-M MULTI (NG Biotech, Guipry, France), an immunochromatographic assay targeting CTX-M type ESBLs, demonstrated a sensitivity of 87.5% and specificity of 98.9%, both with turnaround times of 15–45 minutes. The direct ESBL Etest (bioMérieux, Marcy-l'Étoile, France), as evaluated in the same study, required 16–20 hours of incubation and showed a sensitivity of 91.7% and specificity of 100%. Notably, other research cited by Boattini et al. has described modified rapid synergy protocols using Etest strips that can yield results in under 5 hours with excellent accuracy. Another approach, direct disk diffusion testing performed according to the European Committee on Antimicrobial Susceptibility Testing guidelines, can provide preliminary susceptibility patterns suggestive of ESBL production more rapidly than conventional methods, though it typically requires overnight incubation, and interpretation can be complex (20). In contrast, our direct Cica-beta test provides a result in approximately 16 minutes, demonstrating a significant advantage in speed over methods requiring prolonged incubation, while showing a competitive accuracy profile against other rapid assays.

Furthermore, MALDI-TOF MS-based approaches have been reported for detecting β-lactamase activity by analyzing antibiotic hydrolysis directly from positive blood culture samples (21, 22). While innovative, these techniques have potential limitations. The reported diagnostic performance of this MALDI-based method, when applied directly to blood cultures, includes a sensitivity of 95.8% and a specificity of 93.3% (21), although its accuracy is potentially limited by a noted difficulty in differentiating ESBL from AmpC β-lactamase activity (22). Additionally, the workflow requires a multi-step process with a turnaround time of 3090 minutes (21, 22).

Rapid molecular panels, such as the BioFire FilmArray BCID2 panel used as a comparator in this study, offer the advantage of detecting specific resistance genes like *bla*CTX-M within approximately 1 hour. Multicenter evaluations have confirmed the high accuracy of the BCID2 panel for *bla*CTX-M detection, reporting positive percent agreement rates of up to 98.5% (23–25). However, a significant limitation is that these molecular panels primarily detect the most common ESBL genes (mainly the CTX-M group) and may miss other ESBL types (e.g., some TEM or SHV variants) or other resistance mechanisms like AmpC hyperproduction, which can also confer resistance to extended-spectrum cephalosporins (26, 27). Additionally, the cost per test and initial instrument investment for such molecular systems can be substantial, as detailed in our cost analysis (see Table S2). Our direct Cica-beta method, while detecting the phenotype of ESBL activity rather than specific genes, provides a cost-effective, rapid alternative or complement in settings where broad molecular testing is not feasible.

A recent systematic review and network meta-analysis on the clinical utility of rapid diagnostic tests (28) demonstrated that their combination with antimicrobial stewardship programs significantly reduced mortality compared to conventional blood culture alone or in combination with stewardship (odds ratios [OR]: 0.72 and 0.78, respectively). Additionally, shorter hospital stays (OR: 0.91) and reduced time to optimal therapy (29 h) were observed, suggesting that these advantages can substantially improve patient treatment outcomes. In addition, a retrospective cohort study was conducted in the US using a hospital database focused on patients whose blood cultures tested positive for *E. coli*, *K. pneumoniae*, *K. oxytoca*, and *P. mirabilis* (29). The study evaluated the effect of phenotype-desirable antimicrobial therapy (PDAT), a method that rapidly identifies pathogen susceptibility or resistance to antimicrobials and enables the early administration of optimal therapy. Compared to delayed PDAT, implementing early PDAT significantly reduced 30-day readmission rates by 20%, along with improving clinical outcomes, as measured by the desirability of the outcome ranking analysis. We believe that the direct Cica-beta test has the potential to further enhance the effectiveness of such programs.

In facilities where MALDI-TOF MS has already been implemented, incorporating the direct Cica-beta test into workflows for direct bacterial identification from positive blood culture bottles has the potential to streamline rapid diagnostics. Furthermore, its simple, kit-based design eliminates the need for complex reagent preparation and offers a long shelf life, enhancing its ease of use and practical implementation in a routine clinical laboratory setting. From an economic perspective, the direct Cica-beta test pathway appears to offer a highly cost-effective alternative to broad molecular panels. The estimated direct cost per sample for our rapid phenotypic workflow—which includes the Sepsityper kit (approximately JPY 500), the Cica-beta test strip (JPY 145), and associated technician hands-on time (approx. 6 minutes total, equating to JPY 225 at an estimated rate of JPY 2,250 /hour)—totals approximately JPY 870 (USD ~5.80). This contrasts sharply with the FilmArray BCID2 panel, where the reagent cost alone is JPY 16,000. Including its hands-on time (approx. 3 minutes, JPY 112.5), the direct cost per test is approximately JPY 16,113 (USD ~107.4).

While a comprehensive health-economic analysis must also consider factors like instrument amortization and annual maintenance costs (in our institution, approximately JPY 500,000–950,000 for the MALDI Biotyper and JPY 83,000 for the FilmArray system), the substantially lower direct per-test cost of the Cica-beta pathway makes it an attractive strategy. This is particularly relevant for laboratories already equipped with MALDI-TOF MS, positioning the direct Cica-beta test as a rapid, accurate, and economically prudent tool for improving the management of patients with gram-negative bacteremia.

Ultimately, these advantages in laboratory workflow and cost are most meaningful for their potential impact on patient care. The rapidity and high diagnostic accuracy of the direct Cica-beta test are valuable for prospectively guiding appropriate antimicrobial therapy. While routine ESBL confirmation is less emphasized by recent CLSI guidelines, early knowledge of likely ESBL production in patients with bacteremia remains crucial for timely clinical decision-making. The clinical relevance of this is highlighted by our finding, reported in the Results, that a substantial portion of patients with ESBL bacteremia (40.7%, 11/27) were on an initial empiric regimen that lacked activity against ESBL producers. Our direct method, by providing results approximately 48 hours faster than conventional methods, offers a critical opportunity to switch to an effective therapy far more quickly. This proactive therapeutic adjustment, enabled by rapid diagnostics, has the potential to prevent the clinical consequences of inappropriate initial treatment and improve patient outcomes, as suggested by studies linking timely, targeted therapy to reduced mortality and shorter hospital stays (7–10).

This study has several limitations. First, as a single-center study conducted in Japan, our findings may not be fully generalizable to other geographic regions with different epidemiologies of resistance mechanisms, underscoring the need for future multicenter validation. Second, the study population was predominantly composed of *E. coli*, which was isolated from 78.0% (85/109) of the cases. While this reflects the common etiology of gram-negative bacteremia, the performance of the direct Cica-beta test for less frequent species was not robustly evaluated; the single discordant result involving *K. oxytoca* (ESBL-producing) highlights the potential for species-specific challenges. For instance, in this case, neither the direct Cica-beta test nor the FilmArray system detected ESBL activity, and further analysis using the Cica Genius ESBL Genotype Detection Kit 2 did not detect common ESBL genotypes. The *K. oxytoca* strain in this case may have harbored a chromosomal K1 gene, leading to hyperproduction of K1 β-lactamase, which can confer resistance to extended-spectrum cephalosporins (30). Such strains, along with AmpC-overproducing strains, have previously been reported to pose a risk of misclassification with the Cica-beta test (15), highlighting the need to consider these possibilities when interpreting results. Third, detailed molecular characterization of the β-lactamase genes was not performed, leaving genotype-phenotype correlation as an area for future research. Fourth, this study focused on ESBL detection, and the ability of the method to detect or differentiate other enzymes, such as metallo-beta-lactamases, remains an area for future investigation. Finally, the visual, subjective nature of the colorimetric interpretation is an inherent limitation that is subject to inter-observer variability, which could be mitigated in the future by developing standardized comparison tools or automated reading systems.

In conclusion, the direct Cica-beta test may be a cost-effective and accurate method that complements existing rapid diagnostic protocols. It has the potential to improve patient outcomes by enabling the early initiation of appropriate antimicrobial therapy. This study introduces the utility of a novel direct testing approach that is substantially faster than conventional methods. To further enhance the practical utility of the direct Cica-beta test, future studies should focus on large-scale, multicenter investigations and explore its applicability to various bacterial species.

## Data Availability

The data supporting this study are available from the corresponding author upon reasonable request.

## SUPPLEMENTAL MATERIAL

Tables S1 to S3 spectrum.01112-25-s0001.pdf Overview of patient backgrounds and clinical characteristics, and detailed comparison of reagent costs, and MIC distributions of various antimicrobial agents against ESBL-producing and non-ESBL-producing isolates.

ASM does not own the copyrights to Supplemental Material that may be linked to, or accessed through, an article. The authors have granted ASM a non-exclusive, world-wide license to publish the Supplemental Material files. Please contact the corresponding author directly for reuse.
