# Supplementary material for: Evaluation of a direct method for detecting extended-spectrum β-lactamase using the Cica-beta test on positive blood culture bottles
Source: Microbiol Spectr. 2025 Oct 8;13(11):e01112-25. doi: 10.1128/spectrum.01112-25 (PMC12584670; doi:10.1128/spectrum.01112-25)
Supplement: Tables S1 to S3 — Overview of patient backgrounds and clinical characteristics, and detailed comparison of reagent costs, and MIC distributions of various antimicrobial agents against ESBL-producing and non-ESBL-producing isolates. [file spectrum.01112-25-s0001.pdf]

Supplementary Table S1. Overview of patient backgrounds and clinical characteristics

Variables	Total (n = 109)
Age (years), median (IQR)	81 (74–87)
Male, n (%)	55 (50.5)
Antimicrobial pre-dose in the immediate past	18 (16.5)
Immunosuppressive agents, n (%)	17 (15.6)
Nosocomial infection, n (%)	24 (22.0)
ICU admission, n (%)	5 (4.6)
Blood cultures collected within the first 24 hours after symptom onset, n (%)	92 (84.4)
qSOFA, median (IQR)	1 (0–2)
Pitt bacteremia score, median (IQR)	1 (0–3)
Charlson comorbidity index, median (IQR)	3 (2–4)

IQR, Interquartile Range; ICU, Intensive Care Unit; qSOFA, quick Sequential Organ Failure Assessment

Supplementary Table S2. Detailed comparison of reagent costs

Test Kit	Total Tests	Total Cost (JPY)	Cost per Test (JPY)
Cica-beta test	40	5,800	145
FilmArray	30	480,000	16,000

JPY, Japanese yen

A Cica-beta test kit (Kanto Chemical Co., Inc. Tokyo, Japan), costing JPY 5,800, can be used to conduct 40 tests, resulting in a per-test cost of JPY 145. In comparison, the FilmArray system (BioFire Diagnostics, Salt Lake City, UT, USA) is priced at JPY 480,000 for 30 tests with a per-test cost of JPY 16,000, resulting in a reagent-based cost difference of JPY 15,855/test.

Supplementary Table S3. Minimum inhibitory concentration (MIC) distributions of various antimicrobial agents against ESBL-producing and non-ESBL-producing isolate

Sample ID	Species Identified (MALDI)	Direct Cica-beta	Colony Cica-beta	FilmArray CTX-M	ESBL Confirmatory Test	CMZ MIC (μg/mL)	CAZ MIC (μg/mL)	CTRX MIC (μg/mL)	CFPM MIC (μg/mL)	IPM/CS MIC (μg/mL)	MEPM MIC (μg/mL)	AZT MIC (μg/mL)
3624611	*Escherichia coli*	Negative	Negative	Negative	Non-ESBL	<=16	<=1	<=1	<=2	<=1	<=0.12	<=4
541651	*Escherichia coli*	Negative	Negative	Negative	Non-ESBL	<=16	<=1	<=1	<=2	<=1	<=0.12	<=4
3667069	*Escherichia coli*	Negative	Negative	Negative	Non-ESBL	<=16	<=1	<=1	<=2	<=1	<=0.12	<=4
8233850	*Klebsiella pneumoniae*	Negative	Negative	Negative	Non-ESBL	<=16	<=1	<=1	<=2	<=1	<=0.12	<=4
5871787	*Klebsiella pneumoniae*	Negative	Negative	Negative	Non-ESBL	<=16	<=1	<=1	<=2	<=1	<=0.12	<=4
5217987	*Escherichia coli*	Negative	Negative	Negative	Non-ESBL	<=16	<=1	<=1	<=2	<=1	<=0.12	<=4
1169366	*Klebsiella pneumoniae*	Negative	Negative	Negative	Non-ESBL	<=16	<=1	<=1	<=2	<=1	<=0.12	<=4
4060799	*Klebsiella pneumoniae*	Negative	Negative	Negative	Non-ESBL	<=16	<=1	<=1	<=2	<=1	<=0.12	<=4
2743544	*Escherichia coli*	Negative	Negative	Negative	Non-ESBL	<=16	<=1	<=1	<=2	<=1	<=0.12	<=4
6174861	*Escherichia coli*	Negative	Negative	Negative	Non-ESBL	<=16	<=1	<=1	<=2	<=1	<=0.12	<=4
1608959	*Escherichia coli* (ESBL)	Positive	Positive	Positive	ESBL	8	>8	>2	>16	<=0.5	<=0.12	>8
8283983	*Escherichia coli*	Negative	Negative	Negative	Non-ESBL	<=16	<=1	<=1	<=2	<=1	<=0.12	<=4
2483785	*Escherichia coli*	Negative	Negative	Negative	Non-ESBL	<=16	<=1	<=1	<=2	<=1	<=0.12	<=4
6152858	*Escherichia coli*	Negative	Negative	Negative	Non-ESBL	<=16	<=1	<=1	<=2	<=1	<=0.12	<=4
6170689	*Escherichia coli*	Negative	Negative	Negative	Non-ESBL	<=16	<=1	<=1	<=2	<=1	<=0.12	<=4
5787182	*Escherichia coli*	Negative	Negative	Negative	Non-ESBL	<=16	<=1	<=1	<=2	<=1	<=0.12	<=4
1015005	*Escherichia coli* (ESBL)	Positive	Positive	Positive	ESBL	<=4	4	>2	>16	<=0.5	<=0.12	>8
2807038	*Escherichia coli*	Negative	Negative	Negative	Non-ESBL	<=16	<=1	<=1	<=2	<=1	<=0.12	<=4
315942	*Escherichia coli*	Negative	Negative	Negative	Non-ESBL	<=16	<=1	<=1	<=2	<=1	<=0.12	<=4
340514	*Escherichia coli* (ESBL)	Positive	Positive	Positive	ESBL	<=4	4	>2	>16	<=0.5	<=0.12	>8
1660784	*Escherichia coli* (AmpC)	Negative	Negative	Negative	Non-ESBL	<=16	<=1	<=1	<=2	<=1	<=0.12	<=4
4394529	*Escherichia coli*	Negative	Negative	Negative	Non-ESBL	<=16	<=1	<=1	<=2	<=1	<=0.12	<=4
5661918	*Klebsiella pneumoniae*	Negative	Negative	Negative	Non-ESBL	<=16	<=1	<=1	<=2	<=1	<=0.12	<=4
3935504	*Escherichia coli*	Negative	Negative	Negative	Non-ESBL	<=16	<=1	<=1	<=2	<=1	<=0.12	<=4
2295096	*Klebsiella pneumoniae*	Negative	Negative	Negative	Non-ESBL	<=16	<=1	<=1	<=2	<=1	<=0.12	<=4
4987228	*Escherichia coli* (ESBL)	Positive	Positive	Positive	ESBL	<=4	8	>2	>16	<=0.5	<=0.25	>8
975126	*Klebsiella pneumoniae*	Negative	Negative	Negative	Non-ESBL	<=16	<=1	<=1	<=2	<=1	<=0.12	<=4
2168909	*Escherichia coli* (ESBL)	Positive	Positive	Positive	ESBL	<=4	8	>2	16	<=0.5	<=0.25	>8
8010899	*Proteus mirabilis*	Negative	Negative	Negative	Non-ESBL	<=16	<=1	<=1	<=2	N/R	<=0.12	<=4
6013369	*Escherichia coli*	Negative	Negative	Negative	Non-ESBL	<=16	<=1	<=1	<=2	<=1	<=0.12	<=4
2549993	*Escherichia coli*	Negative	Negative	Negative	Non-ESBL	<=16	<=1	<=1	<=2	<=1	<=0.12	<=4
6179799	*Klebsiella pneumoniae*	Negative	Negative	Negative	Non-ESBL	<=16	<=1	<=1	<=2	<=1	<=0.12	<=4
3889518	*Escherichia coli* (ESBL)	Positive	Positive	Positive	ESBL	<=4	>8	>2	>16	<=0.5	<=0.25	>8
5541251	*Escherichia coli*	Negative	Negative	Negative	Non-ESBL	<=16	<=1	<=1	<=2	<=1	<=0.12	<=4
3487589	*Escherichia coli*	Negative	Negative	Negative	Non-ESBL	<=16	<=1	<=1	<=2	<=1	<=0.12	<=4
3066028	*Escherichia coli*	Negative	Negative	Negative	Non-ESBL	<=16	<=1	<=1	<=2	<=1	<=0.12	<=4
3630695	*Escherichia coli* (ESBL)	Positive	Positive	Positive	ESBL	<=4	>8	>2	>16	<=0.5	<=0.25	>8
870675	*Escherichia coli*	Negative	Negative	Negative	Non-ESBL	<=16	<=1	<=1	<=2	<=1	<=0.12	<=4
1286353	*Proteus mirabilis*	Negative	Negative	Negative	Non-ESBL	<=16	<=1	<=1	<=2	N/R	<=0.12	<=4
8057785	*Klebsiella pneumoniae*	Negative	Negative	Negative	Non-ESBL	<=16	<=1	<=1	<=2	<=1	<=0.12	<=4
6182325	*Escherichia coli*	Negative	Negative	Negative	Non-ESBL	<=16	<=1	<=1	<=2	<=1	<=0.12	<=4
3499336	*Escherichia coli*	Negative	Negative	Negative	Non-ESBL	<=16	<=1	<=1	<=2	<=1	<=0.12	<=4
4006851	*Escherichia coli* (ESBL)	Positive	Positive	Positive	ESBL	<=4	>8	>2	>16	<=0.5	<=0.25	>8
5087618	*Escherichia coli*	Negative	Negative	Negative	Non-ESBL	<=16	<=1	<=1	<=2	<=1	<=0.12	<=4
8280302	*Escherichia coli*	Negative	Negative	Negative	Non-ESBL	<=16	<=1	<=1	<=2	<=1	<=0.12	<=4
2559737	*Escherichia coli* (ESBL)	Positive	Positive	Positive	ESBL	<=4	8	>2	4	<=0.5	<=0.25	8
5880608	*Escherichia coli*	Negative	Negative	Negative	Non-ESBL	<=16	<=1	<=1	<=2	<=1	<=0.12	<=4
8640539	*Escherichia coli* (ESBL)	Positive	Positive	Positive	ESBL	<=4	>8	>2	>16	<=0.5	<=0.25	>8
795768	*Klebsiella pneumoniae*	Negative	Negative	Negative	Non-ESBL	<=16	<=1	<=1	<=2	<=1	<=0.12	<=4
6184338	*Escherichia coli*	Negative	Negative	Negative	Non-ESBL	<=16	<=1	<=1	<=2	<=1	<=0.12	<=4
6184827	*Escherichia coli*	Negative	Negative	Negative	Non-ESBL	<=16	<=1	<=1	<=2	<=1	<=0.12	<=4
6091478	*Klebsiella oxytoca*	Negative	Negative	Negative	Non-ESBL	<=16	<=1	<=1	<=2	<=1	<=0.12	<=4
3977439	*Escherichia coli*	Negative	Negative	Negative	Non-ESBL	<=16	4	<=1	<=2	<=1	<=0.12	>16
8378267	*Escherichia coli*	Negative	Negative	Negative	Non-ESBL	<=16	<=1	<=1	<=2	<=1	<=0.12	<=4
5017841	*Klebsiella pneumoniae*	Negative	Negative	Negative	Non-ESBL	<=16	<=1	<=1	<=2	<=1	<=0.12	<=4
6129877	*Escherichia coli*	Negative	Negative	Negative	Non-ESBL	<=16	<=1	<=1	<=2	<=1	<=0.12	<=4
4220346	*Proteus mirabilis*	Negative	Negative	Negative	Non-ESBL	<=16	<=1	<=1	<=2	N/R	<=0.12	<=4
4813023	*Escherichia coli*	Negative	Negative	Negative	Non-ESBL	<=16	<=1	<=1	<=2	<=1	<=0.12	<=4
822799	*Escherichia coli* (ESBL)	Positive	Positive	Positive	ESBL	<=4	8	>2	>16	<=0.5	<=0.25	>8
6171757	*Escherichia coli*	Negative	Negative	Negative	Non-ESBL	<=16	<=1	<=1	<=2	<=1	<=0.12	<=4
2239423	*Escherichia coli* (ESBL)	Positive	Positive	Positive	ESBL	<=4	>8	>2	>16	<=0.5	<=0.25	>8
3930976	*Escherichia coli*	Negative	Negative	Negative	Non-ESBL	<=16	<=1	<=1	<=2	<=1	<=0.12	<=4
764489	*Escherichia coli*	Negative	Negative	Negative	Non-ESBL	<=16	<=1	<=1	<=2	<=1	<=0.12	<=4
537235	*Klebsiella pneumoniae*	Negative	Negative	Negative	Non-ESBL	<=16	<=1	<=1	<=2	<=1	<=0.12	<=4
4246453	*Escherichia coli*	Negative	Negative	Negative	Non-ESBL	<=16	<=1	<=1	<=2	<=1	<=0.12	<=4
2070800	*Escherichia coli*	Negative	Negative	Negative	Non-ESBL	<=16	<=1	<=1	<=2	<=1	<=0.12	<=4
205207	*Escherichia coli*	Negative	Negative	Negative	Non-ESBL	<=16	<=1	<=1	<=2	<=1	<=0.12	<=4
1572607	*Escherichia coli* (ESBL)	Positive	Positive	Positive	ESBL	<=4	>8	>2	>16	<=0.5	<=0.25	>8
938674	*Escherichia coli* (ESBL)	Positive	Positive	Positive	ESBL	<=4	<=1	>2	>16	<=0.5	<=0.25	2
3876115	*Escherichia coli*	Negative	Negative	Negative	Non-ESBL	<=16	<=1	<=1	<=2	<=1	<=0.12	<=4
110234	*Escherichia coli*	Negative	Negative	Negative	Non-ESBL	<=16	<=1	<=1	<=2	<=1	<=0.12	<=4
3980554	*Klebsiella pneumoniae*	Negative	Negative	Negative	Non-ESBL	<=16	<=1	<=1	<=2	<=1	<=0.12	<=4
180590	*Escherichia coli* (ESBL)	Positive	Positive	Positive	ESBL	<=4	>8	>2	>16	<=0.5	<=0.25	>8
1990010	*Klebsiella pneumoniae* (ESBL)	Positive	Positive	Positive	ESBL	<=4	>8	>2	>16	<=0.5	<=0.25	>8
4278044	*Escherichia coli*	Negative	Negative	Negative	Non-ESBL	<=16	<=1	<=1	<=2	<=1	<=0.12	<=4
6185228	*Escherichia coli*	Negative	Negative	Negative	Non-ESBL	<=16	<=1	<=1	<=2	<=1	<=0.12	<=4
687770	*Escherichia coli* (ESBL)	Positive	Positive	Positive	ESBL	<=4	>8	>2	>16	<=0.5	<=0.25	>8
1957439	*Escherichia coli* (ESBL)	Positive	Positive	Positive	ESBL	<=4	<=1	>2	2	<=0.5	<=0.25	<=1
63003	*Escherichia coli*	Negative	Negative	Negative	Non-ESBL	<=16	<=1	<=1	<=2	<=1	<=0.12	<=4
79998	*Escherichia coli*	Negative	Negative	Negative	Non-ESBL	<=16	<=1	<=1	<=2	<=1	<=0.12	<=4
4614033	*Escherichia coli*	Negative	Negative	Negative	Non-ESBL	<=16	<=1	<=1	<=2	<=1	<=0.12	<=4
6069132	*Escherichia coli*	Negative	Negative	Negative	Non-ESBL	<=16	<=1	<=1	<=2	<=1	<=0.12	<=4
1804879	*Escherichia coli* (ESBL)	Positive	Positive	Positive	ESBL	<=4	8	>2	>16	<=0.5	<=0.25	>8
3331982	*Escherichia coli*	Negative	Negative	Negative	Non-ESBL	<=16	<=1	<=1	<=2	<=1	<=0.12	<=4
319979	*Klebsiella pneumoniae*	Negative	Negative	Negative	Non-ESBL	<=16	<=1	<=1	<=2	<=1	<=0.12	<=4
2787660	*Escherichia coli* (ESBL)	Positive	Positive	Positive	ESBL	<=4	>8	>2	>16	<=0.5	<=0.25	>8
2217610	*Escherichia coli* (ESBL)	Positive	Positive	Positive	ESBL	<=4	>8	>2	>16	<=0.5	<=0.25	>8
6138810	*Escherichia coli* (ESBL)	Positive	Positive	Positive	ESBL	<=4	8	>2	>16	<=0.5	<=0.25	>8
4830286	*Escherichia coli*	Negative	Negative	Negative	Non-ESBL	<=16	<=1	<=1	<=2	<=1	<=0.12	<=4
5794789	*Escherichia coli*	Negative	Negative	Negative	Non-ESBL	<=16	<=1	<=1	<=2	<=1	<=0.12	<=4
6195973	*Escherichia coli*	Negative	Negative	Negative	Non-ESBL	<=16	<=1	<=1	<=2	<=1	<=0.12	<=4
1631807	*Escherichia coli* (ESBL)	Positive	Positive	Positive	ESBL	<=4	8	>2	16	<=0.5	<=0.25	>8
5559659	*Escherichia coli*	Negative	Negative	Negative	Non-ESBL	<=16	<=1	<=1	<=2	<=1	<=0.12	<=4
6195295	*Proteus mirabilis*	Negative	Negative	Negative	Non-ESBL	<=16	<=1	<=1	<=2	N/R	<=0.12	<=4
6197064	*Escherichia coli*	Negative	Negative	Negative	Non-ESBL	<=16	<=1	<=1	<=2	<=1	<=0.12	<=4
3914325	*Escherichia coli*	Negative	Negative	Negative	Non-ESBL	<=16	<=1	<=1	<=2	<=1	<=0.12	<=4
6197342	*Escherichia coli*	Negative	Negative	Negative	Non-ESBL	<=16	<=1	<=1	<=2	<=1	<=0.12	<=4
6197419	*Escherichia coli*	Negative	Negative	Negative	Non-ESBL	<=16	<=1	<=1	<=2	<=1	<=0.12	<=4
386476	*Escherichia coli* (ESBL)	Positive	Positive	Positive	ESBL	<=4	<=1	>2	2	<=0.5	<=0.25	2
1405309	*Escherichia coli*	Negative	Negative	Negative	Non-ESBL	<=16	<=1	<=1	<=2	<=1	<=0.12	<=4
2191179	*Escherichia coli* (ESBL)	Positive	Positive	Positive	ESBL	8	>8	>2	>16	<=0.5	<=0.25	>8
3028520	*Proteus mirabilis*	Negative	Negative	Negative	Non-ESBL	<=16	<=1	<=1	<=2	N/R	<=0.12	<=4
3128810	*Escherichia coli*	Negative	Negative	Negative	Non-ESBL	<=16	<=1	<=1	<=2	<=1	<=0.12	<=4
	Escherichia coli (ESBL)	Negative	Negative	Negative	ESBL	<=16	<=1	>32	4	<=1	<=0.12	>16
4020522	*Klebsiella pneumoniae*	Negative	Negative	Negative	Non-ESBL	<=16	<=1	<=1	<=2	<=1	<=0.12	<=4
4896115	*Escherichia coli*	Negative	Negative	Negative	Non-ESBL	<=16	<=1	<=1	<=2	<=1	<=0.12	<=4
5646235	*Escherichia coli*	Negative	Negative	Negative	Non-ESBL	<=16	<=1	<=1	<=2	<=1	<=0.12	<=4
6192092	*Escherichia coli* (ESBL)	Positive	Positive	Positive	ESBL	<=4	8	>2	>16	<=0.5	<=0.25	>8
6194672	*Klebsiella pneumoniae*	Negative	Negative	Negative	Non-ESBL	<=16	<=1	<=1	<=2	<=1	<=0.12	<=4
	Klebsiella oxytoca	Negative	Negative	Negative	Non-ESBL	<=16	<=1	<=1	<=2	<=1	<=0.12	<=4
8004082	*Klebsiella oxytoca*	Negative	Negative	Negative	Non-ESBL	<=16	<=1	<=1	<=2	<=1	<=0.12	<=4

MALDI: Matrix-Assisted Laser Desorption/Ionization

ESBL: Extended-Spectrum β-Lactamase

CMZ: Cefmetazole

CAZ: Ceftazidime

CTRX: Ceftriaxone

CFPM: Cefepime

IPM: Imipenem

MEPM: Meropenem

AZT: Aztreonam

MIC: Minimum Inhibitory Concentration
